# Factors Influencing Surgical Choices in Breast Cancer Treatment in India: A Comparative Study of Breast-Conserving Surgery vs Mastectomy

**DOI:** 10.7759/cureus.66825

**Published:** 2024-08-13

**Authors:** Sakshi Dubey, Krishnanand Krishnanand, Yogeshwar Shukla, Pratibha Sharma, Snehasish Tripathy, Priya S Kushwah

**Affiliations:** 1 General Surgery, LN Medical College and Research Center, Bhopal, IND; 2 Dental Research Cell, Dr. D.Y. Patil Dental College and Hospital, Dr. D.Y. Patil Vidyapeeth, Pune, IND; 3 General Surgery, LN Medical College and JK Hospital, Bhopal, IND

**Keywords:** cancer, shared decision making, breast-conserving surgery, mastectomy, breast cancer

## Abstract

Background

Breast-conserving surgery (BCS) can make breast cancer treatment less disfiguring and more aesthetically acceptable for women. However, very few patients in India chose to undergo BCS surgery despite eligibility. Therefore, this study aims to explore the factors influencing the surgical choice in the treatment of breast cancer in India.

Materials and methods

This prospective study was conducted at a tertiary care hospital in Central India. Women having stage I/ II breast cancer diagnosis with a tumor size <5 cm were considered. A detailed self-designed questionnaire was used. A chi-square test with a significance level (p-value <0.05) was applied.

Results

Out of 40 females, 80% (n = 32) chose modified radical mastectomy (MRM), whereas 20% (n = 8) opted for BCS. The primary motivations to undergo MRM included concern about cancer recurrence (30%, n = 12), desire to avoid the adverse effects of radiation therapy (25%, n = 10), and fear of radiation therapy (20%, n = 8). Surgeons play a dominant role in determining surgical options, with 80% of MRM cases following the surgeon's recommendation. A significant association was observed between surgical options, education, economic status, locality, and family history (p<0.001). Changes in decision-making regarding the type of surgery after admission to the hospital were significant (p<0.001) after counseling.

Conclusions

The choice between breast conservation and mastectomy is influenced by sociodemographic factors, personal views, and surgeons’ recommendations. Thus, these factors must be considered in preoperative counseling to help patients make informed choices.

## Introduction

Breast cancer is the prevailing form of malignancy [1,2]. India reported 2,21,757 incidences of breast cancer in 2020 which is projected to reach 2,32,832 by 2025 [2,3]. According to projections, breast cancer is expected to account for 15% of the overall cancer burden in the country by 2025 [3]. For many years, modified radical mastectomy (MRM) has been the most commonly used surgical method to treat breast cancer [4]. Recently, the use of breast-conserving surgery (BCS) along with radiotherapy has become widely accepted for the treatment of breast cancer. Advancements in surgical techniques, oncoplastic treatments, and radiation therapy technologies have resulted in equal survival rates between BCS, followed by irradiation and mastectomy. This has made breast cancer treatment less disfiguring and more aesthetically acceptable for women [5-7]. However, the prevalence of BCS is comparatively low in Asian countries [5]. The United States reported a rate of 64.5% for women with early-stage breast cancer who underwent BCS, whereas in Singapore, 31% of breast carcinoma patients underwent BCS [8,9]. In India, only 11.3% of patients chose to undergo BCS procedure despite their eligibility [10].

Studies conducted in low-resource settings have attributed the remarkably low rates of BCS to various patient factors and non-patient factors. Most breast cancer cases are diagnosed at later stages in low- and middle-income countries (LMICs) in comparison to high-income countries (HICs) [9-11]. Various reasons linked to infrastructure (such as availability, accessibility, and pricing), patients' socio-demographic factors, a dearth of surgeons with specific training in BCS, patients' concerns and worries about recurrence and radiation, and variation in surgeons' recommendation for surgeries have also been identified as contributing to the preference for mastectomy over BCS [4,10,12-14]. The concentration of adjuvant radiation facilities and interdisciplinary teams in metropolitan regions creates resource imbalance [4]. In addition, 75% of cancer expenses in India are paid directly by individuals, resulting in delayed detection and treatment [12]. The obligatory inclusion of adjuvant radiation to breast-conserving surgery contributes further to this load. In India, societal attitudes towards women's health and physician-patient dynamics may contribute to the high rates of mastectomy, indicating a significant gap in meeting the needs for breast reconstruction [10]. There is a notable lack of understanding about patient perceptions regarding breast reconstruction, especially regarding their actual needs and satisfaction within the Indian context.

To address this gap, the aim of this study is to determine the factors influencing the surgical choice in the treatment of breast cancer among Indian women. This study seeks to better understand the complicated interplay between patient sociodemographic variables, therapeutic choices, and physician recommendations in the treatment of breast cancer in India. The findings from this study will provide insights that could influence healthcare practices and policy formulation, ultimately contributing to more informed and patient-centred care practices.

## Materials and methods

Study design, study setting and procedure

This prospective study enrolled 40 patients and spanned 16 months, from July 2022 to November 2023. It aimed to explore the reasons behind the selection of surgical options and the changes in these choices during different phases of counselling for patients diagnosed with stage I or II breast cancer.

The study was carried out at a tertiary care facility in central India, where patients were closely monitored from initial registration to operation planning. Surgeons counselled patients on several surgical alternatives, including a detailed explanation of the benefits and drawbacks of each. Counselling sessions were held in the hospital's outpatient department (OPD), and patients were supervised by a medical team led by the same surgeon in the ward following admission until the final surgical plan was finalized. An identical crew member conducted the re-counselling within the ward.

Participants: inclusion and exclusion criteria

Patients with initial stages of breast cancer (stages I or II) with tumours smaller than 5 cm were included. The study excluded patients with metastatic or locally advanced breast cancer.

Data collection tool and procedure

A detailed self-designed questionnaire was used to document demographic information and evaluate factors influencing patients' surgical decisions. These factors included patient-related variables (e.g., age, marital status, education, economic status, family history), the individual responsible for making the treatment decision (self/husband/self + husband/other relative), and personal concerns associated with the surgical option. The data collection followed the informed consent of each participant, who was also provided with an information leaflet detailing the study's objectives and ensuring the confidentiality of their identifying information. Diagnostic assessments included fine-needle aspiration cytology (FNAC), chest X-ray, abdominal and pelvic ultrasounds, and mammography or breast ultrasounds based on the patient's age. Patients aged >40 years underwent bilateral mammography.

Analysis

The data was entered into Microsoft excel sheet and cleaned. Following that, the statistical analysis was conducted in Statistical Package for Social sciences (SPSS) software for Windows, version 28.0 (IBM Corp., Armonk, NY, USA). Descriptive statistics such as frequency and percentage were used to describe data. To assess the significance of differences in responses between the two surgical groups (modified radical mastectomy vs. breast-conserving surgery) based on the variables, a chi-square test was applied with a level of significance set at a p-value <0.05.

Ethical consideration

The study was approved by the ethical review board of LN Medical College and JK Hospital (LNMC & RC/Dean/ 2023/ethics/045). Participants were informed of their right to voluntary participation, right to withdraw at any point of time and risk-benefit associated with participation in research. Written and verbal consent was taken from all participants before participation in the study.

## Results

Socio-demographic characteristics of participants

**Figure 1:**
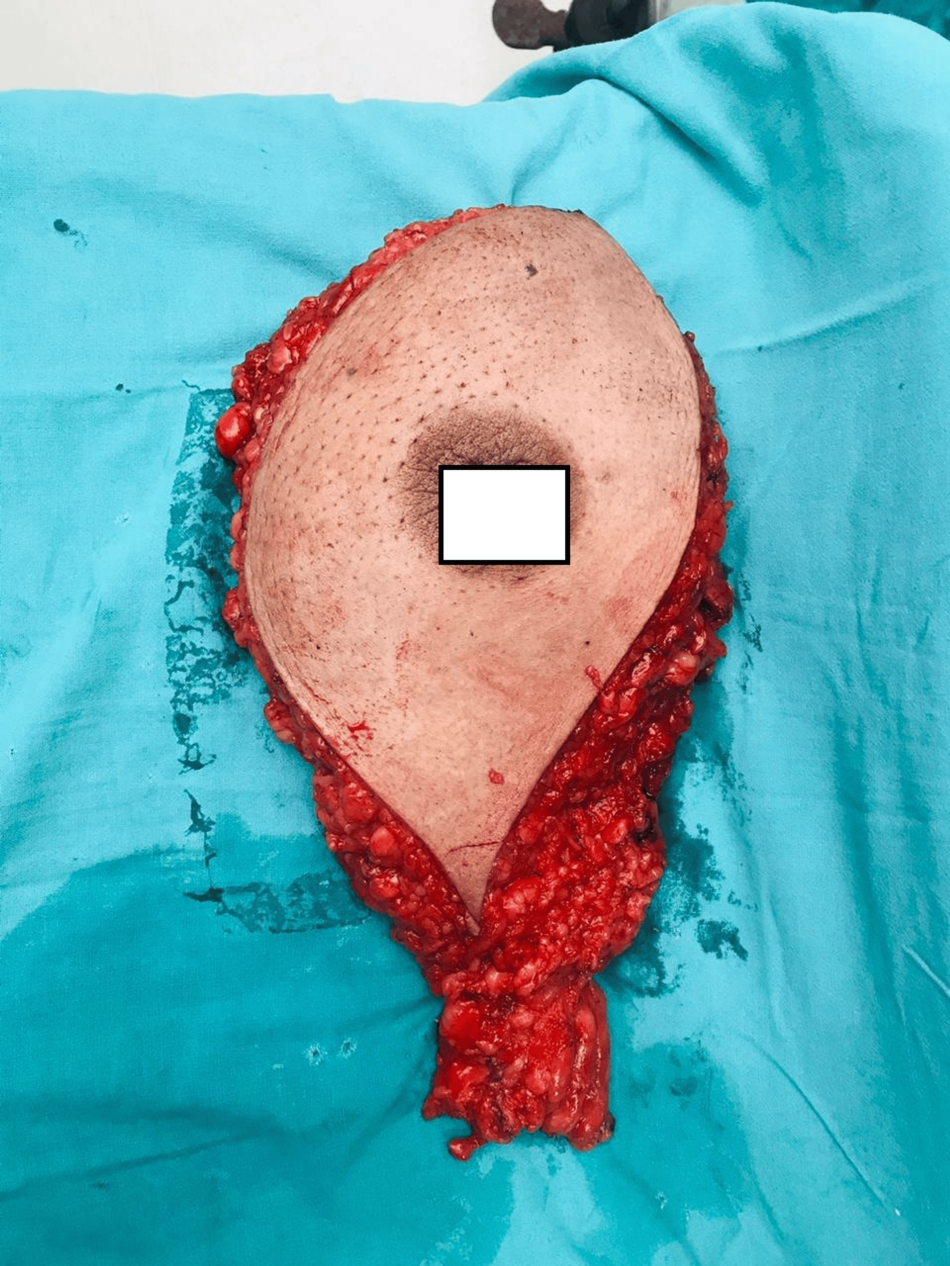
Young patient with early-stage carcinoma breast undergone modified radical mastectomy with nipple-areola complex removed

**Figure 2:**
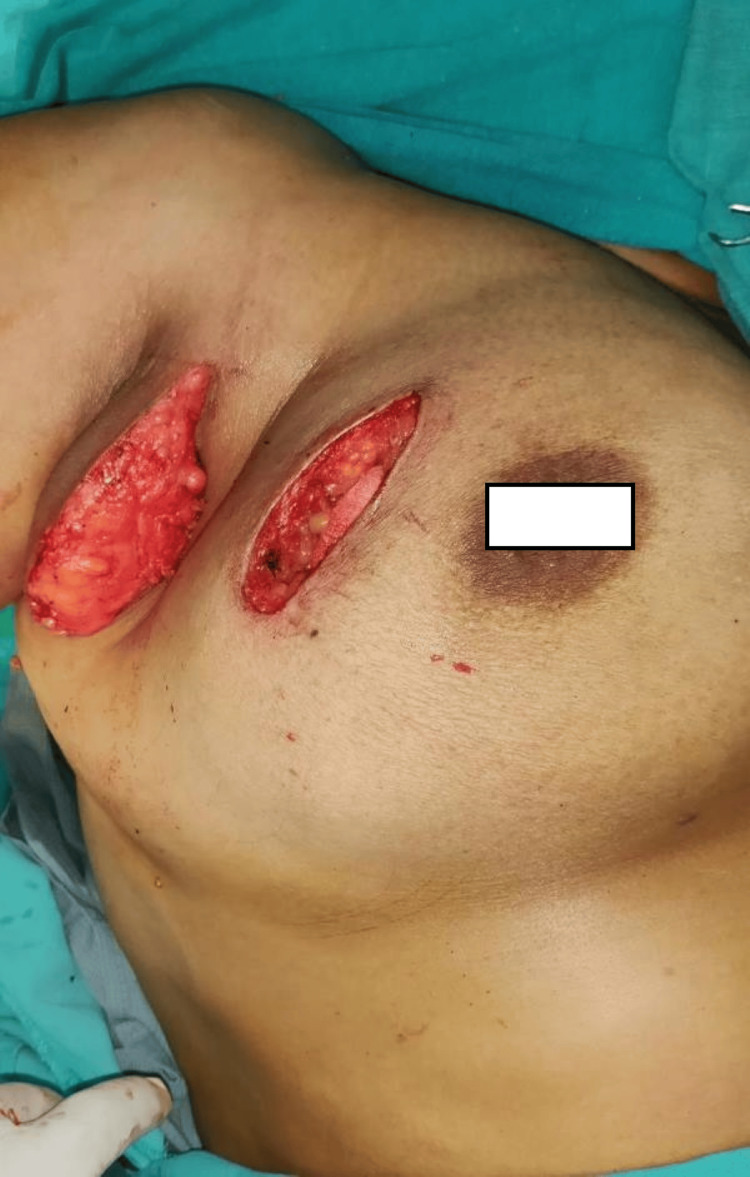
Axillary dissection

**Figure 3:**
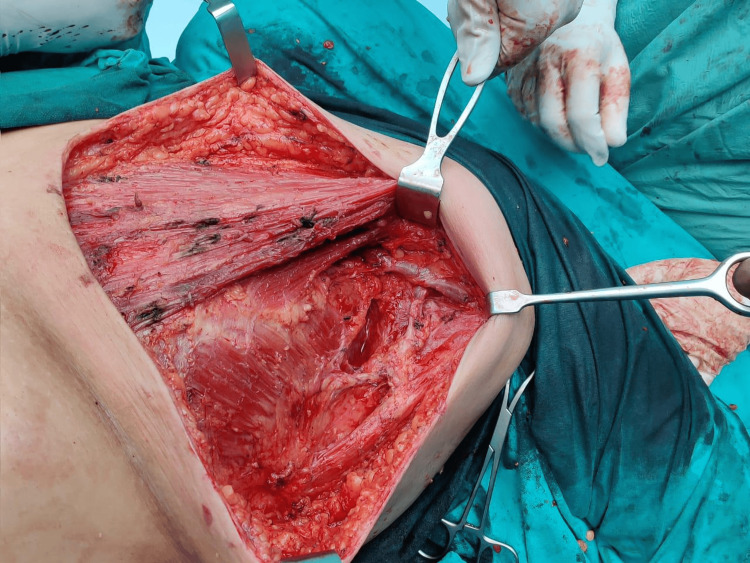
Intraoperative image of breast conservative surgery with modified radical mastectomy

**Figure 4:**
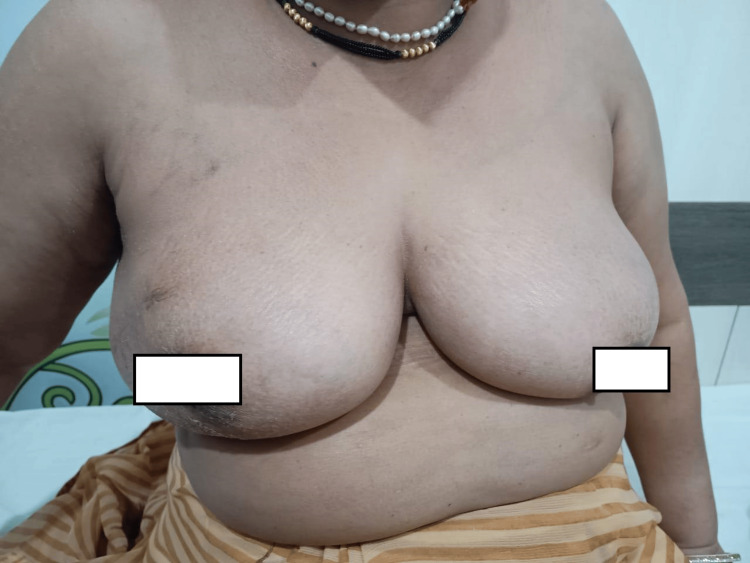
Breast conservative surgery: post-operative minimal scarring with intact nipple-areola complex

The study included all female participants (n=40). None of the included participants were breastfeeding at the time of participation in the study. Among them, 79.1% (n=32) chose MRM, whereas 20.9% (n=8) chose BCS. It is worth noting that eight women changed their minds about MRM and switched to BCS after getting hospital counselling (Figures 1-4).

Only four of the 40 females were unmarried, with the other 36 married. The age group gap included 30% (n = 12) females under 40 and 70% (n = 28) over 40. Only 17.5% (n = 6) of the 40 female participants exhibited co-occurring conditions, including hypertension (HTN), diabetes mellitus (DM), combination (HTN and DM), and asthma. All of the female participants were literate; among them, 30% (n = 12) individuals had completed primary schooling, 25% (n = 10) had completed high school, 32.5% (n = 13) had done graduate studies, and 2.5% (n = 5) had achieved post-graduation degrees of education. The percentage of females in urban areas was 45% (n = 18), while that in rural areas was 55% (n = 22). The study population included individuals with diverse economic statuses: 55% (22) were non-earning dependents, 30% (12) were earning dependents, and 15% (6) were financially independent.

Factors influencing decision-making for mastectomy vs BCS

A significant association was observed with educational, economic status, locality, family history, surgery-related concerns, and decision factors. Individuals with higher education levels - graduation (n = 3) and post-graduation (n = 5) - chose BCS, while those with lower education (school and high school) exclusively opted for MRM. Similarly, economic dependence played a role, with non-earning dependent individuals overwhelmingly choosing MRM, and economically independent individuals (n = 8) choosing BCS. The Chi-square tests for these categories were highly significant (p<0.001). Patients from urban areas were found to be significantly associated with opting for BCS (p<0.001). A strong family history of breast cancer was predominantly associated with choosing MRM (n = 29), with significant statistical support (p<0.001).

Fear of radiation (radiophobia) (25%, n = 10), mitigating the adverse effects of radiation therapy (25%, n = 10), and fear of cancer recurrence (carcinophobia) (30%, n = 12) were also significantly linked to choosing MRM. Conversely, concerns about body image post-mastectomy (20%, n = 8) led to a preference for BCS. These findings were significant with a p-value of 0.00.

Changes in decision-making regarding the type of surgery after admission to the hospital were significant (p<0.001), with 8 (n = 20%) patients who changed their decision opting for BCS after counseling. The decision-making process was also influenced by who was involved; decisions taken jointly by patients and their husbands (42.5%, n = 17) and husbands with surgeons (n = 15, 37.5%) were more likely to result in choosing BCS compared to decisions made solely by the surgeon, however, this finding was non-significant. There was no statistically significant difference in the choice of the surgical method based on age (p = 0.80), marital status (p = 0.54) and co-morbidity status (p = 0.54) (Table 1).

**Table 1:** Comparison of surgery group across the study group A, radiophobia; B, mitigating the adverse effects of radiation therapy; C, carcinophobia; D, mastectomy-related body image distress; DM: diabetes mellitus; HTN: hypertension; MRM: modified radical mastectomy; BCS: breast-conserving surgery.

Narration	MRM	BCS	Total	Chi-square	P-value	
Age group						
< = 40 years	10	2	12 (30)	0.1		0.80
> 40 years	22	6	28 (70)			
Marital status						
Married	28	8	36 (90)	0.4		0.54
Unmarried	4	0	4 (10)			
Comorbid conditions						
No comorbidity	26	7	33 (82.5)	0.17		0.68
Either DM/HTN or both	6	1	7 (17.5)			
Educational status						
Primary School	12	0	12 (30)	25.57		< 0.001
High school	10	0	10 (25)			
Graduation	10	3	13 (32.5)			
Post-graduation	0	5	5 (12.5)			
Economic status						
Non-earning dependent	22	0	22 (55)	29.59		< 0.001
Earning dependent	10	2	12 (30)			
Independent	0	6	6 (15)			
Locality						
Urban	10	8	18 (45)	28.8	< 0.001	
Rural	22	0	22 (55)			
Family history of CA breast						
Yes	29	1	30 (75)	16.87		<0.001
No	3	7	10 (25)			
Reason for procedure (MRM/BCS)						
A (MRM)	10	0	10 (25)	40		0.00
B (MRM)	10	0	10 (25)			
C (MRM)	12	0	12 (30)			
D (BCS)	0	8	8 (20)			
Decision change or not						
No	32	0	32 (80)	33.99		<0.001
Yes	0	8	8 (20)			
Decision taken by						
Surgeon	8	0	8 (20)	2.94		0.22
Surgeon +husband	12	3	15 (37.5)			
Patient +husband	12	5	17 (42.5)			

## Discussion

Although evidence demonstrates that breast-conserving surgery (BCS) improves the quality of life for eligible patients, many women who qualify for BCS choose mastectomy. Our research found that treatment decisions are influenced by a complicated combination of demographic characteristics, worries of cancer recurrence, and decision-making processes. Understanding these characteristics allows healthcare professionals to deliver tailored education to support patients in making informed treatment decisions.

The first crucial finding to consider is that education level, economic dependency, and locality all have a substantial impact on the decision-making process about breast cancer surgery. Individuals with higher education levels are more likely to choose BCS than MRM, a tendency mirrored by data from other studies [15-17]. This choice may originate from the fact that patients with poor educational backgrounds frequently struggle to understand the benefits and drawbacks of both surgical alternatives, reducing their confidence in making independent decisions. Furthermore, some patients continue to perceive mastectomy as the sole trustworthy option for breast cancer therapy, believing that a more thorough procedure delivers better results [15,18]. Conversely, individuals with higher education levels tend to express their opinions more freely and are more likely to discuss alternative surgical options. Similarly, economic independence plays a crucial role in surgical decision-making. Financially independent people have greater authority over the decision-making process and are more confident in selecting the surgeries they choose. Another factor is the importance of aesthetic appearance in professional settings. Economically independent women, who might be concerned about job security, may prefer BCS due to its better aesthetic outcomes [17]. This preference highlights the complex interplay between education, economic status, and personal choices in breast cancer surgery. Similarly, Frisell et al. [19] reported a higher incidence of BCS among women of higher education, and socioeconomic status. They also verified that women who have a lower socioeconomic level (SES) frequently have tumors that are at an advanced stage, which could further affect the decision to have a mastectomy.

Another important element influencing the decision to have breast cancer surgery is one's locality. Radiation therapy is usually necessary following breast-conserving surgery (BCS), therefore the requirement for many hospital stays as well as the possible hardship of travel, lodging, and job absenteeism can greatly influence the surgical choice. The nearest intra-operative radiation therapy (IORT) centers are located 30 km and 5 km away from the study area. This availability might have influenced patients' decisions by making breast-conserving surgery (BCS) with IORT a more convenient and appealing option compared to modified radical mastectomy (MRM) for some patients who have no accessibility issues. The logistical challenges associated with BCS can contribute to the preference for mastectomy among women from distant areas and those with low economic status. Similar findings have been noted in studies globally [18,20]. For instance, Dicks et al. [18] reported that to minimize the inconvenience of making frequent trips to medical institutions, women were 1.15 times more likely to select mastectomy for every 40 minutes that they spent driving or traveling. In India, the lack of patient-assisted travel schemes, especially for rural patients, exacerbates this issue. The direct and indirect costs incurred from travel and accommodation strongly influence the decision-making process, often leading to a preference for mastectomy over BCS due to the reduced need for frequent follow-up visits.

Although individual belief factors are among the most significant influences on women's decisions regarding breast cancer surgery, they are also the most difficult to study and often the least examined. Our research identified that a positive family history, concern about recurrence, radiophobia, and its adverse effects were significant and body image concerns are the main factors that influence patients’ decision for surgical choice. Studies by Raghavendra et al. [21] and Dicks et al. [18] also found higher rates of mastectomy among women with a family history of breast cancer, attributed to their heightened self-perceived risk of developing the disease and its recurrence. On the contrary, Yuksel et al. [17] and Moiel et al. [22] reported no association between a positive family history and the choice of surgery. Instead, they identified complex factors such as fear of tumor recurrence, potential secondary interventions due to positive surgical margins, and concerns about radiotherapy post-BCS as the main reasons influencing this decision [17]. These findings underscore the intricate nature of decision-making based on personal beliefs, highlighting the need for large-scale studies to thoroughly understand these influences.

The majority of research has indicated that age is a significant factor in determining the choice between mastectomy and BCS with older patients in Asia, specifically those aged >60 years, showing a greater preference for mastectomy over breast conservation prioritizing the physical impact of radiation therapy on cosmetic aspects [15,23]. Contrarily, the current investigation revealed no disparity in age between the two patient groups. The average age along with the standard deviation of patients who underwent MRM was 49.5 ± 11.6.

Shared decision-making in treatment is a novel idea in patient-centred care that allows patients and physicians to agree on treatment based on their values and preferences [24]. However, recent research indicates that the decision-making process for breast cancer treatment is complex and often challenging for women, with decisions typically made quickly, often during the first meeting with a surgeon [24]. While some patients prefer to take an active role, others defer to their surgeons [25]. In concordance with this, our study also found that surgeons play a dominant role in determining surgical options, with 80% of MRM cases following the surgeon's recommendation and only 20% opting for BCS. In affluent nations, about 40% to 80% of women make autonomous decisions [26], whereas, in our study, surgeons made most decisions, followed by joint decisions with husbands (11.9%) and decisions by patients with their husbands (9.6%). This could be because women in rural areas often have lower education and financial independence, leading to less priority on their health issues and passivity in decision-making. Factors such as fear of recurrence, limited radiotherapy facilities, and the unavailability of rapid pathological exams in smaller hospitals further influence the preference for mastectomy [15]. In a qualitative study [18], surgeons observed that women had a poor grasp of the comparability of surgical alternatives for early-stage breast cancer, as well as a strong desire to have extensive surgery to alleviate their anxieties and increase their chances of survival. However, counseling of women was found to be significantly associated with a change in the decision as noted in other studies as well [27]. This suggests that providing information in various forms such as through written information or audio-visual means and allowing patients to review it when less distressed can improve understanding [18]. Measures like national leaflets, assigned breast contact nurses, and information folders could also be beneficial [19]. Within our custom-designed questionnaire, we included a section to assess patients' comprehensive understanding of the process following counseling. All patients who chose to undergo mastectomy with immediate reconstruction (MRM) responded positively. However, this positive response may be attributed to a lack of understanding of cancer biology and limited financial resources, which influenced their decision to opt for MRM instead of breast-conserving surgery (BCS).

The current study employed a prospective design, providing patients who met the eligibility requirements for BCS with a genuine free choice between BCS and mastectomy, provided they were educated about the significance of radiation as a component of treatment and that there was no variation in the result between the two operations. However, this study has several limitations. The study was limited to a single institution and had a relatively small sample size which limits its generalizability to a larger population. Moreover, the sociodemographic and personal factors influencing the decision can significantly vary across regions and cultural context. Thus, larger longitudinal and experimental studies are needed in India to further explore these findings. Besides, structural and systemic factors that might influence the decision-making process for breast cancer surgery were not explored. Additionally, psychological factors, including patients' mental health and emotional responses to their diagnosis and treatment options, were not examined. Furthermore, the study did not investigate how patients' perceptions of body image and sexuality impact their surgical choices. These limitations highlight the need for further research to comprehensively understand the various factors influencing surgical decisions in breast cancer treatment.

Our findings have certain policy and practical implications. This highlights the importance of increased preoperative counseling, in which surgeons recognize and address sociodemographic aspects and personal perspectives, resulting in more informed decision-making. Utilizing a patient-centered strategy that takes into account socioeconomic status and cultural views can boost satisfaction and post-operative results. Educating healthcare providers in cultural competency and strong communication skills is critical for effective patient engagement. Developing healthcare policies that guarantee all patients have equal access to information and options can help to eliminate disparities. Strategies such as community outreach, educational campaigns, and support groups tailored for distinct populations are suggested.

## Conclusions

The choice between breast conservation and mastectomy is a complex decision-making process which could be influenced by sociodemographic factors, personal views, and surgeons’ recommendations. Thus, these factors must be taken into consideration in preoperative counseling. Care teams must ensure that patients make treatment decisions based on informed personal preferences and values. Future large-scale research should focus on understanding various factors influencing surgical choices to support informed, personalized treatment decisions.
